# Detection of β-lactam resistance genes in Gram-negative bacteria from positive blood cultures using a microchip-based molecular assay

**DOI:** 10.3389/fcimb.2025.1597700

**Published:** 2025-06-03

**Authors:** Vittorio Ivagnes, Flavio De Maio, Ilaria Baccani, Alberto Antonelli, Giulia Menchinelli, Roberto Rosato, Giordana Cafaro, Giulia Santarelli, Federico Falletta, Tiziana D’Inzeo, Maurizio Sanguinetti, Teresa Spanu, Giulia De Angelis, Gian Maria Rossolini, Brunella Posteraro

**Affiliations:** ^1^ Dipartimento di Scienze Biotecnologiche di Base, Cliniche Intensivologiche e Perioperatorie, Università Cattolica del Sacro Cuore, Rome, Italy; ^2^ Dipartimento di Scienze di Laboratorio ed Ematologiche, Fondazione Policlinico Universitario A. Gemelli Istituto di Ricovero e Cura a Carattere Scientifico (IRCCS), Rome, Italy; ^3^ Dipartimento di Medicina Sperimentale e Clinica, Università di Firenze, Florence, Italy; ^4^ Struttura Organizzativa Dipartimentale (SOD) Microbiologia e Virologia, Azienda Ospedaliera Universitaria Careggi, Florence, Italy; ^5^ Unità Operativa “Medicina di Precisione in Microbiologia Clinica”, Direzione Scientifica, Fondazione Policlinico Universitario A. Gemelli Istituto di Ricovero e Cura a Carattere Scientifico (IRCCS), Rome, Italy

**Keywords:** antimicrobial resistance, β-lactamase, GNR microchip assay, Gram-negative bacteria, molecular detection, positive blood cultures

## Abstract

**Background:**

Accurate detection of β-lactam resistance genes in bloodstream infections is critical for guiding antimicrobial therapy. This study evaluates the Alifax Gram-negative resistance (GNR) microchip assay for detecting β-lactam resistance genes directly from positive blood cultures (PBCs) for Gram-negative (GN) bacteria, including Enterobacterales, *Pseudomonas aeruginosa*, and *Acinetobacter baumannii*.

**Methods:**

Simulated (n=146) and clinical (n=106) GN-PBC samples were tested for *bla*_KPC_, *bla*_VIM_, *bla*_NDM_, *bla*_IMP_, *bla*_OXA-23_-like, *bla*_OXA-48_-like, *bla*_SHV_-ESBL, *bla*_CTX-M-1/9_ group, and *bla*_CMY-2_-like genes using the GNR microchip assay. Whole-genome sequencing (WGS) served as the reference assay for simulated samples and, selectively, for clinical samples. The bioMérieux BioFire Blood Culture Identification 2 (BCID2) panel assay was used as a comparator for clinical samples.

**Results:**

The GNR microchip assay correctly identified 203 (99.5%) of 204 β-lactam resistance genes in simulated samples. One sample tested false negative for a *bla*_SHV_-ESBL gene but true positive for a *bla*_KPC_ gene. In clinical samples, GNR results were concordant with BCID2 for 113 (100%) of 113 genes included in both assays. Additionally, the GNR assay detected bla*_CMY-2_*-like (n=6), *bla*_OXA-23_-like (n=5), and *bla*_SHV_-ESBL (n=2), which are not targeted by BCID2, all confirmed by WGS. In two β-lactam-resistant *P. aeruginosa* samples but negative by the GNR assay, WGS confirmed the absence of acquired β-lactam resistance genes, suggesting alternative resistance mechanisms.

**Conclusion:**

The GNR microchip assay demonstrated high concordance and broader β-lactam resistance gene coverage compared to BCID2, supporting its potential role in routine diagnostics. Further validation in larger, prospective studies is warranted.

## 1 Introduction

Bloodstream infections (BSIs) represent a critical category of microbial infections where the rapid identification of causative pathogens is essential for timely and effective clinical decision-making (Peri et al., 2022). Accelerated diagnostic methods play a pivotal role in enabling the prompt initiation of targeted antimicrobial therapy (Lamy et al., 2020), particularly in patients at risk of sepsis, an infectious syndrome associated with alarmingly high morbidity and mortality rates (GBD 2019 Antimicrobial Resistance Collaborators, 2022). Furthermore, the increasing global prevalence of antimicrobial resistance (AMR) in BSIs (Diekema et al., 2019; European Antimicrobial Resistance Collaborators, 2022) underscores the urgent need for diagnostic approaches that not only identify pathogens but also detect key AMR markers. This is particularly relevant for Gram-negative (GN) bacterial species, such as third-generation cephalosporin-resistant and carbapenem-resistant Enterobacterales (GBD 2021 Antimicrobial Resistance Collaborators, 2024; Ikhimiukor et al., 2024), where AMR significantly limits therapeutic options and emphasizes the importance of its early detection.

Although the clinical impact of carbapenemase production is well established, the prevalence and significance of non-carbapenemase-producing extended-spectrum β-lactamase (ESBL)-producing Enterobacterales remain less defined. The Centers for Disease Control and Prevention (CDC) reported that in 2017, there were an estimated 197,400 cases of ESBL-producing Enterobacterales among hospitalized patients in the United States, resulting in approximately 9,100 deaths (CDC, 2025). Additionally, a study from Finland observed that the annual proportion of ESBL-producing *E. coli* among blood isolates increased from 2.4% to 8.6% in males and from 1.6% to 6.4% in females over a 12-year period (Ilmavirta et al., 2023). Regarding ESBL types, Castanheira et al. (2021) noted that the global dominance of CTX-M-type β-lactamases has largely supplanted SHV-type ESBLs, reflecting a shift in epidemiology. However, SHV-ESBLs are often encoded by self-transmissible plasmids that frequently harbor resistance genes to other antibiotic classes, suggesting that their contribution to β-lactam resistance might still be significant, especially in specific geographical contexts (Liakopoulos et al., 2016).

Conventional culture-based methods remain the cornerstone of BSI diagnosis (Lamy et al., 2020), but their inherent slowness—requiring 24–72 hours for pathogen identification and antimicrobial susceptibility testing (AST) (Miller et al., 2018)—poses challenges in time-sensitive clinical settings. To address these limitations, molecular diagnostic assays such as the BioFire Blood Culture Identification 2 (BCID2) panel (bioMérieux, Marcy l’Étoile, France) (Berinson et al., 2021) have been introduced to accelerate pathogen identification and resistance gene detection directly from positive blood cultures (PBCs) (Peker et al., 2018). These assays provide actionable results within hours, allowing clinicians to initiate targeted therapy earlier than culture-based methods. However, their limitations, including high costs and restricted coverage of detectable pathogens and AMR markers, highlight the ongoing need for innovative solutions in molecular diagnostics.

This study reports on the evaluation of a novel molecular assay, the Gram-negative resistance (GNR) microchip (Alifax S.r.l., Polverara, PD, Italy), which received the Conformité Européene (CE)-*in vitro* device (IVD) certification in 2022 for the detection of clinically relevant AMR genes in GN bacterial species from PBCs. The assay specifically targets β-lactam resistance genes, including those encoding ESBLs and carbapenemases, and was evaluated using both simulated and clinical PBCs.

## 2 Methods

### 2.1 Study setting and samples

This study was conducted at the clinical microbiology laboratory of the Fondazione Policlinico Universitario A. Gemelli IRCCS, a large tertiary-care teaching hospital in Rome, Italy, over a one-year period (April 2023 to March 2024). To evaluate the Alifax GNR microchip assay, simulated (n=146) and clinical (n=106) positive blood culture (PBC) samples for GN bacterial organisms were used. Samples were obtained after incubation of BacT/Alert FA (aerobic) or FN (anaerobic) blood culture (BC) bottles (bioMérieux) and subsequent positive flagging by the BacT/Alert Virtuo BC automated system (bioMérieux). The aerobic or anaerobic bottle was analyzed depending on which flagged positive first.

**Figure 1:**
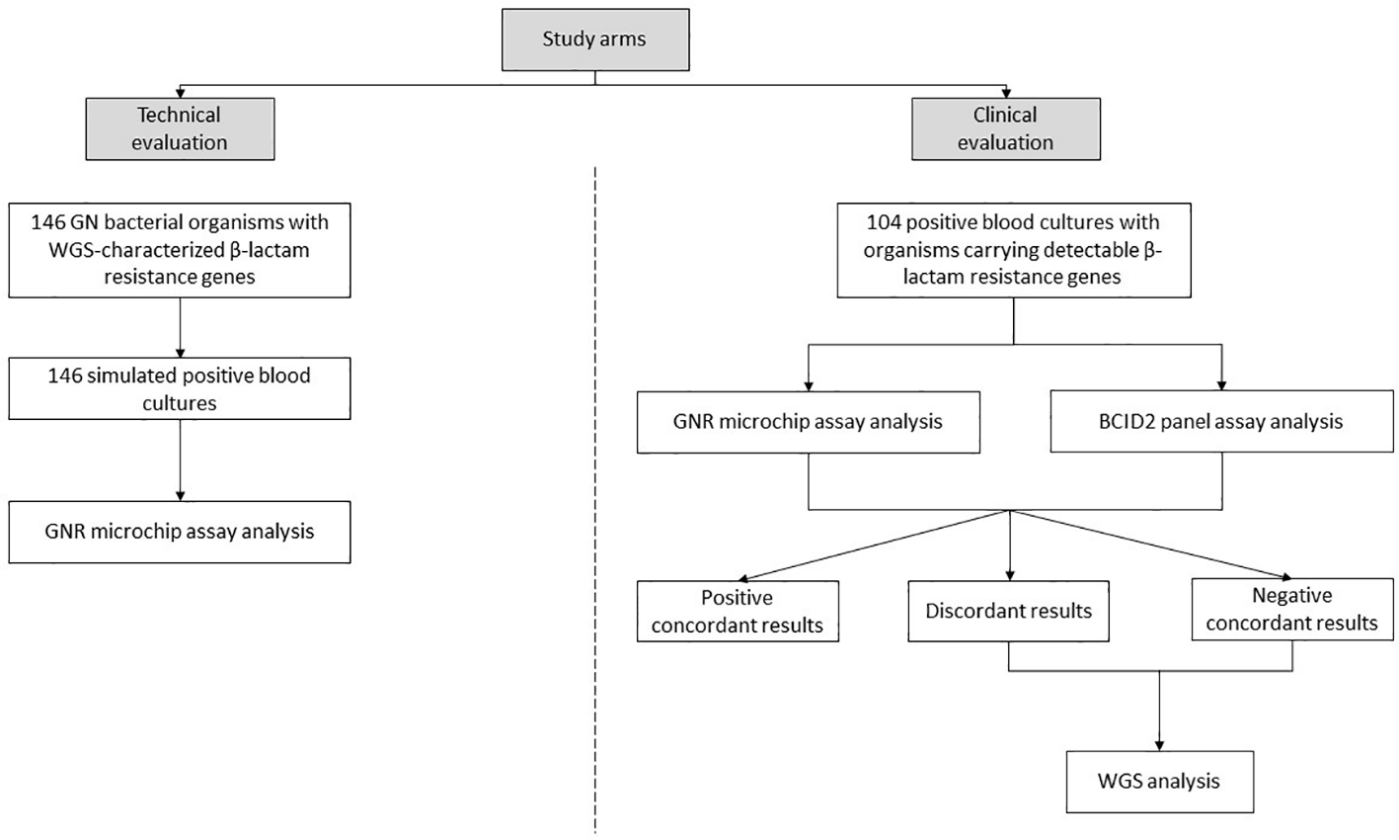
Study design overview. Two sets of positive blood cultures for Gram-negative (GN) bacterial organisms were analyzed to detect β-lactam resistance genes using the GNR microchip assay. In the first set, detection results were compared with whole-genome sequencing (WGS) analysis. In the second set, detection results were compared with the BioFire Blood Culture Identification 2 (BCID2) panel assay and, in specific cases, with WGS analysis.

The study consisted of two arms, as illustrated in **Figure 1**:

- Technical evaluation arm, in which simulated PBC samples were obtained by spiking bacterial cells from isolates (one per bottle) into whole blood according to established procedures (Menchinelli et al., 2019). The 146 bacterial organisms used in the simulation experiments had been characterized through WGS for the presence of β-lactam resistance genes prior to their inclusion in this study. A subset of these organisms had already been described in previous studies (David et al., 2019; Di Pilato et al., 2021, 2022; Giani et al., 2017, 2018), while others were newly sequenced in this study. Details on the sequencing data for all organisms are provided in **Supplementary Table S1**. The selected isolates were chosen to represent a wide range of β-lactamase-encoding genes, with the aim of covering all targets detectable by the GNR microchip assay (with the exception of the CTX-M-2/8 group, which was not represented among available isolates).
- Clinical evaluation arm, in which clinical PBC samples were obtained as part of routine laboratory analysis. If Gram-stain microscopy confirmed the presence of GN bacteria and monomicrobial growth, samples were considered eligible for the inclusion in the study. Aliquots from samples were collected after species identification (via MALDI-TOF mass spectrometry; Bruker Daltonics, Bremen, Germany) (Fiori et al., 2016) and AST (via Vitek 2 system, bioMérieux) (De Angelis et al., 2019). Samples were included in the study based on phenotypic resistance criteria to maximize the detection of β-lactam resistance genes targeted by the GNR microchip assay. Specifically, GN bacterial organisms were selected if their minimum inhibitory concentration (MIC) values for at least one of the third-generation cephalosporins (cefotaxime or ceftazidime) or one of the carbapenems (imipenem or meropenem), as determined by routine Vitek 2 AST results, were above the EUCAST epidemiological cut-off values (ECOFFs) (EUCAST, 2025a). For bacterial species lacking defined ECOFFs, EUCAST resistant breakpoints (EUCAST, 2025b) were used instead. The selected samples were directly analyzed using the already mentioned BioFire BCID2 panel assay, which detects bacterial species and resistance genes, including those encoding carbapenemases (*bla*_IMP_, *bla*_KPC_, *bla*_OXA-48_-like, *bla*_NDM_, *bla*_VIM_), ESBLs (*bla*_CTX-M_), and the colistin resistance protein (*mcr-1*). Only results for the six β-lactam resistance genes were considered in this study.

Aliquots from each PBC bottle were directly analyzed using the GNR microchip assay (described below) or, in cases as specified below, plated on MacConkey and 5% sheep blood tryptic soy agar media (bioMérieux). Overnight-grown isolates were subsequently analyzed via the WGS assay.

### 2.2 GNR microchip assay

The GNR microchip contains all the reagents required for multiplex real-time PCR using the molecular mouse (MM) instrument (Alifax) for qualitative DNA target detection (Alifax, 2024). These targets encompass 13 AMR markers specific to GN bacteria, including *bla*_KPC_, *bla*_VIM_, *bla*_NDM_, *bla*_IMP_, *bla*_OXA-23_-like, *bla*_OXA-48_-like, *bla*_SHV_, *bla*_SHV_-ESBL, *bla*_CTX-M-1/9_ group, *bla*_CTX-M-2/8_ group, *bla*_CMY-2_-like, *mcr-1*, and *mcr-2*. For this study, *mcr-1* and *mcr-2* were excluded as they are not associated with β-lactam resistance, while *bla*_SHV_ was excluded despite its relevance to β-lactam resistance, due to its high prevalence across multiple microbial species and limited clinical utility in this context. The GNR microchip assay specifically targets *bla*_SHV_-ESBL, as variants with this phenotype have greater clinical relevance compared to non-ESBL variants (Castanheira et al., 2021).

In this study, a 200-μL PBC sample was initially centrifuged at 500×g for 1 minute. The resulting supernatant was transferred into a clean tube and centrifuged at 5000×g for 1 minute. The supernatant was then discarded, and the pellet was resuspended in 1 mL of H_2_O. A 100-μL aliquot of this suspension was mixed with 900-μL of loading solution, and the mixture was thoroughly vortexed. Following the manufacturer’s instructions, a 5-μL volume of the prepared solution was loaded onto the GNR cartridge. At the end of the PCR, the MM instrument software automatically captured and analyzed the fluorescence signals from each well where the PCR reactions occurred. The software generated a graphical output, with positive results indicated by cycle threshold values, defined as the number of cycles required for the fluorescent signal to exceed the threshold, confirming positive detection.

### 2.3 Whole-genome sequencing assay

GN bacterial organisms used for simulated PBC samples had been characterized by WGS before their inclusion in this study, whereas those from clinical PBC samples with discordant results between the GNR microchip assay and the BCID2 panel assay, or with β-lactam-resistant phenotypes but negative GNR microchip assay detection, underwent further WGS analysis. DNA was extracted using the DANAGENE Microbial DNA kit (Danagen-Bioted, Barcelona, Spain), and its concentration and purity were assessed with a NanoDrop One spectrophotometer (Thermo Fisher, Waltham, MA, USA).

Short-read genomic data were generated by preparing DNA libraries with the Illumina DNA Prep kit (Illumina, San Diego, CA, USA) and sequencing them on an Illumina MiSeq DX platform according to the manufacturer’s protocols. Details of the WGS analysis pipeline, including library preparation, sequencing, and bioinformatic workflows, have been described previously (Posteraro et al., 2024). AMR markers, including β-lactam resistance genes and point mutations, were identified using the AMRFinderPlus v.3.11.18 (https://github.com/ncbi/amr) and ABRicate v.1.0.1 (https://github.com/tseemann/abricate) tools.

Accession numbers for the sequencing data of all GN bacterial isolates are provided in **Supplementary Table S1**.

### 2.4 Data analysis

The GNR microchip assay was evaluated by comparing its results to those obtained with the WGS assay for simulated samples and, for clinical samples, to those from the BCID2 panel assay. Results were expressed as the proportion of β-lactam resistance genes correctly detected (positive by both assays) or not detected (negative by both assays). Discordant results were further analyzed by WGS. Statistical analyses were performed using GraphPad Prism (GraphPad Software, San Diego, CA, USA).

## 3 Results

We studied GN bacterial organisms from simulated (n=146) and clinical (n=106) PBC samples, corresponding to the technical and clinical evaluations of the GNR microchip assay (**Figure 1**). All organisms exhibited phenotypic antimicrobial profiles suggestive of acquired ESBL/AmpC and/or carbapenemase-encoding genes, based on MIC values exceeding the EUCAST-established ECOFFs or resistant breakpoints for key β-lactams, including third-generation cephalosporins (cefotaxime and ceftazidime) and carbapenems (imipenem and meropenem).

As shown in **Table 1**, using WGS as the reference assay, the GNR microchip assay correctly detected at least one β-lactam resistance gene in all 146 (100%) organisms from simulated samples. This corresponded to a total of 203 β-lactam resistance genes, including 84 encoding ESBL/AmpC-type enzymes (39 CMY-2-like, 38 CTX-M-1/9 group, and 7 SHV-ESBL) and 119 encoding carbapenemase-type enzymes (37 KPC, 27 NDM, 16 VIM, 16 OXA-48-like, 13 OXA-23-like, and 10 IMP). Among these, 49 organisms (47 Enterobacterales and 2 *Acinetobacter baumannii*) carried more than one β-lactam resistance gene. Notably, one *K. pneumoniae* isolate was positive for an SHV-ESBL gene (*bla*_SHV-31_) by WGS but was also positive for a KPC-encoding gene, which was correctly detected by the GNR microchip assay.

**Table 1:** Results of GNR microchip assay for simulated GN-PBC samples as compared to the WGS reference assay.

Species (no. of organisms tested)	No. of genes detected by the GNR assay (no. of genes detected by the WGS assay)^a^									
	*bla*_CMY-2_-like	*bla*_CTX-M-1/9_-group	*bla*_KPC_	*bla*_IMP_	*bla*_NDM_	*bla*_OXA-23_-like	*bla*_OXA-48_-like	*bla*_SHV_-ESBL	*bla*_VIM_	Total genes
*K. pneumoniae* (70)	11 (11)	31 (31)	35 (35)	0 (0)	18 (18)	0 (0)	11 (11)	2 (3)^b^	3 (3)	111 (112)
*E. coli* (28)	11 (11)	7 (7)	0 (0)	0 (0)	5 (5)	0 (0)	5 (5)	4 (4)	5 (5)	37 (37)
*P. aeruginosa* (14)	1 (1)	0 (0)	0 (0)	10 (10)	1 (1)	0 (0)	0 (0)	0 (0)	2 (2)	14 (14)
*A. baumannii* (13)	0 (0)	0 (0)	0 (0)	0 (0)	2 (2)	13 (13)	0 (0)	0 (0)	0 (0)	15 (15)
*P. mirabilis* (12)	12 (12)	0 (0)	0 (0)	0 (0)	0 (0)	0 (0)	0 (0)	1 (1)	1 (1)	14 (14)
*C. freundii* (3)	2 (2)	0 (0)	1 (1)	0 (0)	0 (0)	0 (0)	0 (0)	0 (0)	2 (2)	5 (5)
*E. cloacae* (2)	1 (1)	0 (0)	0 (0)	0 (0)	0 (0)	0 (0)	0 (0)	0 (0)	1 (1)	2 (2)
*C. koseri* (1)	0 (0)	0 (0)	1 (1)	0 (0)	0 (0)	0 (0)	0 (0)	0 (0)	0 (0)	1 (1)
*P. monteilii* (1)	0 (0)	0 (0)	0 (0)	0 (0)	0 (0)	0 (0)	0 (0)	0 (0)	1 (1)	1 (1)
*P. stuartii* (1)	1 (1)	0 (0)	0 (0)	0 (0)	1 (1)	0 (0)	0 (0)	0 (0)	0 (0)	2 (2)
*R. ornithinolytica* (1)	0 (0)	0 (0)	0 (0)	0 (0)	0 (0)	0 (0)	0 (0)	0 (0)	1 (1)	1 (1)
Total species (146)	39 (39)	38 (38)	37 (37)	10 (10)	27 (27)	13 (13)	16 (16)	7 (8)	16 (16)	203 (204)

GNR, Gram-negative resistance; GN-PBC, Gram-negative-positive blood culture; WGS, whole-genome sequencing. ^a^ Details on the β-lactam resistance gene variants identified by WGS analysis are provided in **Table 3**. ^b^ This was the only false-negative result observed with the Alifax GNR microchip assay, involving a GN-PBC sample that grew an organism carrying a *bla*_SHV_-_31_ ESBL gene identified by WGS analysis. Therefore, the concordance between the GNR assay and WGS for the total samples was 99.5% (203/204).

As shown in **Table 2**, the GNR microchip assay detected at least one β-lactam resistance gene in 104 (98.1%) of 106 organisms from clinical samples. Excluding 13 genes not detectable by the BCID2 panel assay (6 encoding CMY-2-like, 2 encoding SHV-ESBL, and 5 encoding OXA-23-like; all identified by WGS), this corresponded to a total of 113 β-lactam resistance genes correctly detected by the GNR microchip assay. These genes included 65 encoding ESBL-type enzymes (all CTX-M-1/9 group) and 48 encoding carbapenemase-type enzymes (32 KPC, 7 VIM, 6 NDM, and 3 OXA-48-like). Additionally, two organisms (*P. aeruginosa*, 1.9%) tested negative for ESBL/AmpC- or carbapenemase-encoding genes by the GNR microchip assay. WGS analysis confirmed the absence of acquired β-lactam resistance genes in these isolates, suggesting that alternative mechanisms, such as increased expression of efflux pumps, may explain their β-lactam-resistant phenotype. Six organisms (2 *Escherichia coli*, 1 *Klebsiella pneumoniae*, 1 A*. baumannii*, 1 *Enterobacter cloacae*, and 1 *Citrobacter freundii*) carried more than one β-lactam resistance gene. These genes were concordantly detected by both the GNR microchip assay and the WGS reference assay and, as expected, only partially by the BCID2 panel assay. Details on these genes are provided as **Supplementary Material** (**Supplementary Table S2**).

**Table 2:** Results of GNR microchip assay for clinical GN-PBC samples as compared to the BCID2 panel assay.

Species (no. of organisms tested)	No. of genes detected by the GNR assay (no. of genes detected by the BCID2 assay)^a^									
	*bla*_CMY-2_-like	*bla*_CTX-M-1/9_-group	*bla*_KPC_	*bla*_IMP_	*bla*_NDM_	*bla*_OXA-23_-like	*bla*_OXA-48_-like	*bla*_SHV_-ESBL	*bla*_VIM_	Total genes
*K. pneumoniae* (48)	2 (–)	23 (23)	31 (31)	0 (0)	4 (4)	0 (–)	3 (3)	0 (–)	1 (1)	64 (62)
*E. coli* (40)	3 (–)	38 (38)	1 (1)	0 (0)	0 (0)	0 (–)	0 (0)	0 (–)	0 (0)	42 (39)
*A. baumannii* (5)	0 (–)	0 (0)	0 (0)	0 (0)	1 (1)	5 (–)	0 (0)	0 (–)	0 (0)	6 (1)
*P. aeruginosa* (4)	0 (–)	0 (0)	0 (0)	0 (0)	0 (0)	0 (–)	0 (0)	0 (–)	4 (4)	4 (4)
*P. mirabilis* (3)	0 (–)	3 (3)	0 (0)	0 (0)	0 (0)	0 (–)	0 (0)	0 (–)	0 (0)	3 (3)
*E. cloacae* (2)	0 (–)	1 (1)	0 (0)	0 (0)	0 (0)	0 (–)	0 (0)	2 (–)	1 (1)	4 (2)
*C. freundii* (1)	1 (–)	0 (0)	0 (0)	0 (0)	0 (0)	0 (–)	0 (0)	0 (–)	1 (1)	2 (1)
*P. stuartii* (1)	0 (–)	0 (0)	0 (0)	0 (0)	1 (1)	0 (–)	0 (0)	0 (–)	0 (0)	1 (1)
Total species (104)	6 (–)	65 (65)	32 (32)	0 (0)	6 (6)	5 (–)	3 (3)	2 (–)	7 (7)	126 (113)

GNR, Gram-negative resistance; GN-PBC, Gram-negative-positive blood culture; BCID2, Blood Culture Identification 2. The symbol “–”indicates data that are either unavailable or not applicable, specifically for β-lactam resistance genes, such as *bla*_CMY-2_-like, *bla*_OXA-23_-like, or *bla*_SHV_-ESBL, which are not detectable by the bioMérieux BioFire Blood Culture Identification 2 (BCID2) panel assay. Excluding these cases, the concordance between the GNR assay and BCID2 panel across all samples was 100% (113/113). In these samples (all growing β-lactam-resistant GN organisms), whole-genome sequencing (WGS) identified one or more β-lactam-resistance genes not included in the BCID2 panel; details on the gene variants are provided in **Table 3**. Additionally, the set included 2 β-lactam-resistant *P. aeruginosa* organisms in which the GNR assay did not detect any β-lactam resistance genes. For both organisms, WGS confirmed the absence of acquired β-lactam resistance genes, supporting the GNR assay results.

**Table 3** provides an overview of β-lactam resistance gene variants identified in this study by WGS analysis. Among organisms from simulated PBC samples, the most frequently detected β-lactamase types were CMY-2-like (n=39), CTX-M-1/9 group (n=38), KPC (n=37), and NDM (n=27), collectively accounting for 141 (69.1%) of the 204 β-lactamase-encoding genes identified. The predominant variants within each β-lactamase type were CMY-16 (21/39), CTX-M-15 (35/38), KPC-3 (25/37), and NDM-1 (24/27). For CTX-M variants (Naas et al., 2017), CTX-M-15 was the most prevalent and belongs to the CTX-M-1 group, which also includes CTX-M-32 (one organism) and CTX-M-55 (one organism), while CTX-M-27 was the only representative of the CTX-M-9 group (one organism). For KPC variants (Naas et al., 2017), KPC-3 was the most frequent (25/37) and, along with KPC-2 (three organisms), belongs to the carbapenemase class. In contrast, KPC-31 (four organisms), KPC-66 (two organisms), KPC-49 (one organism), and KPC-50 (one organism) are classified as inhibitor-resistant (IR) extended-spectrum β-lactamases (ESBLs), while KPC-29 (one organism) is classified as an IR carbapenemase, based on the established β-lactamase classification system (Naas et al., 2017).

**Table 3:** Variants of β-lactamases encoded by the resistance genes identified using whole-genome sequencing analysis.^a^.

Simulated positive blood culture samples																																		
CMY-2-like		n		CTX-M-1/9 group		n		IMP		n		KPC		n		NDM		n		OXA-23-like		n		OXA-48-like		n		SHV-ESBL		n		VIM		n
CMY-16		21		CTX-M-15		35		IMP-13		9		KPC-3		25		NDM-1		24		OXA-23		13		OXA-48		9		SHV-12		8		VIM-1		13
CMY-2		5		CTX-M-27		1		IMP-19		1		KPC-31		4		NDM-5		3						OXA-181		6		SHV-31		1		VIM-2		2
CMY-42		5		CTX-M-32		1						KPC-2		3										OXA-244		1								
CMY-6		4		CTX-M-55		1						KPC-66		2																				
CMY-4		1										KPC-29		1																				
CMY-65		1										KPC-49		1																				
CMY-99		1										KPC-50		1																				
CMY-181		1																																
Total		39		Total		38		Total		10		Total		37		Total		27		Total		13		Total		16		Total		9		Total		15
Clinical positive blood culture samples^b^																																		
CMY-2-like	n		CTX-M-1/9 group		n		IMP		n		KPC		n		NDM		n		OXA-23-like		n		OXA-48-like		n		SHV-ESBL		n		VIM		n	
CMY-16	2		CTX-M-15		3						KPC-3		1		NDM-1		1		OXA-23		5						SHV-12		2		VIM-1		3	
CMY-2	1		CTX-M-3		1																													
CMY-4	1																																	
CMY-147	1																																	
CMY-150	1																																	
Total	6		Total		4						Total		1		Total		1		Total		5						Total		2		Total		3	

a The β-lactamases are listed according to the designation of their respective targets included in the Alifax Gram-negative resistance (GNR) microchip assay evaluated in this study. Notably, the CTX-M-1/9 group comprises two distinct subgroups: CTX-M-1 (including CTX-M-15, CTX-M-32, CTX-M-55, and CTX-M-3) and CTX-M-9 (including CTX-M-27). Similarly, the CTX-M-2/8 group (not listed here) consists of two subgroups: CTX-M-2 and CTX-M-8/25. Among the KPC variants detected, KPC-3 and KPC-2 are classified as carbapenemases, whereas KPC-31, KPC-66, KPC-49, and KPC-50 are classified as inhibitor-resistant (IR) extended-spectrum β-lactamases. KPC-29 is classified as an IR carbapenemase.

b Whole-genome sequencing (WGS) analysis was initially planned for three targets (*bla*_CMY-2_-like, *bla*_OXA-23_ *-like*, and *bla*_SHV_-ESBL), which are not included in the bioMérieux BioFire Blood Culture Identification 2 (BCID2) panel assay. The BCID2 assay served as a comparator for the Alifax Gram-negative resistance (GNR) microchip assay for these samples. Additionally, WGS analysis enabled the identification of gene variants for targets (*bla*_CTX-M,_ *bla*_KPC_, *bla*_NDM_, and *bla*_VIM_) also included in the BCID2 panel assay, particularly in samples where the GNR microchip assay detected multiple genes. These results encompass both targets covered by the BCID2 assay and those exclusive to the GNR microchip assay. Consequently, the total results for some targets presented here do not match the totals shown in **Table 2** (see text for details).

## 4 Discussion

This study provides a comprehensive evaluation of the Alifax GNR microchip assay for the detection of β-lactam resistance genes directly from PBC samples. To our knowledge, this is the first study assessing its performance on a large set of PBC samples, including both simulated samples—derived from WGS-characterized isolates—and clinical samples. The findings demonstrate that the GNR assay reliably detects a wide spectrum of β-lactam resistance genes, with a broader target range compared to the BCID2 panel assay, which served as a comparator for clinical samples.

In simulated samples, the GNR assay detected all but one of the β-lactam resistance genes identified by WGS (99.5% concordance). The single discordant case involved an SHV-ESBL gene (*bla*_SHV-31_) that was not detected by the GNR assay, though a coexisting KPC gene was correctly identified. In clinical samples, 98.1% of organisms carried at least one β-lactam resistance gene detected by the GNR assay, and for genes targeted by both assays, results were fully concordant with those of the BCID2 assay. WGS analysis confirmed 13 additional β-lactam resistance genes not included in BCID2, further supporting the broader detection capability of the GNR assay.

The inclusion of *bla*_CMY-2_-like and *bla*_OXA-23_-like, which are absent from the BCID2 assay, enhances the GNR assay’s diagnostic utility. CMY-2 is the most common plasmid-mediated AmpC β-lactamase in Enterobacterales (Pires et al., 2015), while OXA-23 is a key carbapenemase in *A. baumannii* (Koirala et al., 2020), highlighting the clinical relevance of these targets. However, the GNR assay does not include some β-lactam resistance genes relevant for *Acinetobacter* species such as OXA-58 and OXA-24/40 (Evans and Amyes, 2014), which could further enhance its coverage.

WGS analysis in simulated samples provided insights into the distribution of β-lactamase variants, emphasizing the relevance of distinguishing functionally different groups. The classification of KPC enzymes, for instance, is increasingly recognized as clinically significant (Ding et al., 2023). While KPC-3 and KPC-2 function as carbapenemases, other variants such as those found in this study (KPC-31, KPC-66, KPC-49, and KPC-50) exhibit IR-ESBL activity, whereas KPC-29 is classified as an IR-carbapenemase. Notably, KPC-31 and KPC-66 have been linked to resistance to ceftazidime-avibactam (CZA) (Ding et al., 2023), underscoring the need for precise differentiation of β-lactamase variants to inform antimicrobial therapy. Similarly, while the GNR microchip assay effectively detects CTX-M-producing Enterobacterales, its grouping of CTX-M targets into CTX-M-1/9 (and CTX-M-2/8) does not fully account for the clinically relevant distinction between CTX-M-1 and CTX-M-9 subgroups. This differentiation is particularly important given that certain CTX-M variants have been associated with distinct epidemiological trends and β-lactam resistance profiles (Castanheira et al., 2021; Bush and Bradford, 2020). A more refined classification of CTX-M and KPC subtypes in molecular assays could improve the clinical utility of β-lactam resistance detection, enhancing treatment decisions and antimicrobial stewardship efforts.

While this study provides strong technical evaluation of the GNR microchip assay, several limitations should be acknowledged. First, the study design did not include a consecutive clinical sample set but instead enriched for β-lactam-resistant organisms based on phenotypic criteria, limiting the generalizability of findings to routine clinical workflows. Second, WGS was not performed for all clinical PBC samples, leaving some resistance gene profiles incompletely characterized. Third, the study does not assess the potential clinical impact of the assay. Rapid molecular assays, including the GNR microchip assay, are most valuable when they facilitate early targeted therapy adjustments (Mauri et al., 2024), particularly in BSIs where timely intervention is crucial (World Health Organization, 2025). Evaluating whether the assay enables faster clinical decisions, reduces time to appropriate therapy, or improves outcomes would be critical to justify its integration into diagnostic workflows.

Fourth, unlike BCID2, the GNR microchip assay requires an additional Gram stain step to select the appropriate cartridge, which may delay processing and reduce suitability in time-critical settings. Fifth, although the MM system also provides species identification (ID) when combined with the GNID cartridge—designed to detect 15 key GN pathogens including *E. coli*, *K. pneumoniae*, *Proteus* spp., *P. aeruginosa*, *A. baumannii*, *Stenotrophomonas maltophilia*—species-level ID data were not reported. Nonetheless, all isolates, including *K. oxytoca* and *P. aeruginosa*, were correctly identified, contrasting with prior reports of occasional failures in polymicrobial samples (Mauri et al., 2024).

Sixth, we did not report results for an additional 46 samples with β-lactam-susceptible organisms, in which the GNR microchip assay did not detect any β-lactam resistance genes. This is because no WGS analysis was performed on these isolates that would have been necessary to confirm the GNR microchip assay-negative results. Seventh, the assay’s performance in polymicrobial samples was not evaluated, and its ability to resolve mixed resistance profiles (e.g., *E. coli* + *K. pneumoniae*) remains to be determined. Eighth, while potential interference from sample-related inhibitors (e.g., heparin, hemolysis) was not assessed in our simulated PBCs, internal validation by the manufacturer did not reveal significant effects from common inhibitors.

Ninth, although no formal cost-effectiveness analysis was conducted, the combined cost of the GNR and GNID cartridges is below €100, which is lower than the BCID2 panel assay (>€140). In settings where MALDI-TOF MS-based identification is already implemented, pairing it with the GNR microchip assay may offer a focused and cost-effective diagnostic alternative for GN-PBCs. However, this approach may require specific workflow adaptations and staff training. Tenth, while the GNR microchip assay received CE-IVD certification in 2022, the process of certification under the new European IVDR framework is ongoing, and the assay is not currently FDA-cleared. In contrast, the BCID2 panel is FDA-cleared and widely adopted in clinical practice. These regulatory differences may influence the assay’s adoption in different healthcare systems.

Finally, the GNR microchip assay focuses specifically on β-lactam resistance and does not detect resistance markers for other antibiotic classes. Future versions could benefit from expanded gene targets, particularly for non-fermenting GN bacteria (Huang et al., 2024), and the inclusion of point mutations affecting β-lactamase activity (Benhadid-Brahmi et al., 2025). Integration with phenotypic AST systems—such as the Vitek Reveal rapid AST by bioMérieux (Menchinelli et al., 2024)—could enhance clinical utility by offering a broader phenotypic-genotypic resistance overview (Cortes-Lara et al., 2025). Several of these aspects should be carefully evaluated before adopting the assay in specific diagnostic scenarios.

## 5 Conclusion

In conclusion, the GNR microchip assay represents a reliable molecular tool for detecting β-lactam resistance genes from PBC samples, with potential applications in routine diagnostics. Its expanded gene coverage compared to existing panels is a major strength, particularly for AMR determinants not targeted by other assays. However, further validation in larger, prospective clinical cohorts is warranted to establish its full diagnostic value. Future studies should also assess hands-on time, turnaround time, and real-world implementation, including clinical impact and antimicrobial stewardship outcomes.

## Data Availability

The datasets presented in this study can be found in online repositories. The names of the repository/repositories and accession number(s) can be found in the article/**Supplementary Material** (see **Table S1** footnote).

## Supplementary material

Supplementary Table 1 Details of Enterobacterales (n=118), *Pseudomonas* spp. (n=15), and *Acinetobacter baumannii* (n=13) isolates with β-lactam resistance mechanisms characterized by whole-genome sequencing.

Supplementary Table 2 Comparison of detection results across assays for six clinical GN-PBC samples with multiple β-lactam resistance genes identified. GN-PBC, Gram-negative-positive blood culture; GNR, Gram-negative resistance; WGS, whole-genome sequencing; BCID2, Blood Culture Identification 2.
