# Supplementary material for: Detection of β-lactam resistance genes in Gram-negative bacteria from positive blood cultures using a microchip-based molecular assay
Source: Front Cell Infect Microbiol. 2025 Jun 3;15:1597700. doi: 10.3389/fcimb.2025.1597700 (PMC12170645; doi:10.3389/fcimb.2025.1597700)
Supplement: Supplementary Table 1 — Details of Enterobacterales (n=118), Pseudomonas spp. (n=15), and Acinetobacter baumannii (n=13) isolates with β-lactam resistance mechanisms characterized by whole-genome sequencing. [file Table1.pdf]

TABLE S1 Details of Enterobacterales (n=118), *Pseudomonas* spp. (n=15), and *Acinetobacter baumannii* (n=13) isolates with β-lactam resistance mechanisms characterized by whole-genome sequencing.

**Designation**	**Species**	**Sequence Accession Numbers**	**Reference**
FIR 5	*Pseudomonas aeruginosa*	GCA_002136195.1	Giani et al., 2018 [doi:10.1093/jac/dkx453]
FIR 9	*Pseudomonas aeruginosa*	GCA_002135835.1	
FIR 10	*Pseudomonas aeruginosa*	GCA_002135765.1	
FIR 11	*Pseudomonas aeruginosa*	GCA_002135705.1	
FIR 12	*Pseudomonas aeruginosa*	GCA_002135715.1	
FIR 13	*Pseudomonas aeruginosa*	GCA_002136375.1	
FIR 14	*Pseudomonas aeruginosa*	GCA_002135615.1	
FIR 15	*Pseudomonas aeruginosa*	GCA_002135625.1	
FIR 20	*Klebsiella pneumoniae*	SRR14820319	Di Pilato et al., 2022 [doi: 10.1016/S2666-5247(21)00268-8]
FIR 21	*Klebsiella pneumoniae*	SRR12518029	
FIR 22	*Klebsiella pneumoniae*	SRR12518030	
FIR 23	*Klebsiella pneumoniae*	SRR12518033	
FIR 24	*Klebsiella pneumoniae*	SRR12518054	
FIR 25	*Klebsiella pneumoniae*	SRR12518051	
FIR 26	*Klebsiella pneumoniae*	SRR12518043	
FIR 27	*Klebsiella pneumoniae*	SRR12518048	
FIR 28	*Klebsiella pneumoniae*	SRR12518017	
FIR 29	*Klebsiella pneumoniae*	GCA_900512855.1	David et al., 2019 [doi: 10.1038/s41564-019-0492-8]
FIR 31	*Klebsiella pneumoniae*	GCA_900513015.1	
FIR 32	*Klebsiella pneumoniae*	GCA_900513045.1	
FIR 33	*Klebsiella pneumoniae*	GCA_900513255.1	
FIR 34	*Klebsiella pneumoniae*	GCA_900513245.1	
FIR 35	*Klebsiella pneumoniae*	GCA_900513305.1	
FIR 37	*Klebsiella pneumoniae*	GCA_900514915.1	
FIR 38	*Klebsiella pneumoniae*	GCA_900515235.1	
FIR 40	*Klebsiella pneumoniae*	GCA_900514345.1	
FIR 42	*Klebsiella pneumoniae*	GCA_900516165.1	
FIR 43	*Klebsiella pneumoniae*	GCA_900516145.1	
FIR 44	*Klebsiella pneumoniae*	GCA_900516245.1	
FIR 45	*Klebsiella pneumoniae*	GCA_900515615.1	
FIR 46	*Klebsiella pneumoniae*	GCA_900515625.1	
FIR 47	*Klebsiella pneumoniae*	GCA_902705105	
FIR 48	*Klebsiella pneumoniae*	GCA_902703605	Di Pilato et al, 2021 [doi: 10.1093/jac/dkaa431.]

FIR 49	*Klebsiella pneumoniae*	GCA_902705205	
FIR 50	*Klebsiella pneumoniae*	GCA_902704785	
FIR 51	*Klebsiella pneumoniae*	GCA_902704775	
FIR 52	*Proteus mirabilis*	PRJNA1203573	Giani et al., 2017 [doi: 10.2807/1560-7917.ES.2017.22.34.30601]
FIR 53	*Proteus mirabilis*	PRJNA1203573	
FIR 54	*Proteus mirabilis*	PRJNA1203573	
FIR 55	*Proteus mirabilis*	PRJNA1203573	
FIR 56	*Proteus mirabilis*	PRJNA1203573	
FIR 57	*Proteus mirabilis*	PRJNA1203573	
FIR 58	*Proteus mirabilis*	PRJNA1203573	
FIR 59	*Proteus mirabilis*	PRJNA1203573	
FIR 60	*Proteus mirabilis*	PRJNA1203573	
2401G0023	*Klebsiella pneumoniae*	SAMN45881402	This study
2401G0036	*Klebsiella pneumoniae*	SAMN45881432	This study
2401G0037	*Klebsiella pneumoniae*	SAMN45881448	This study
2401G0039	*Klebsiella pneumoniae*	SAMN45881399	This study
2401G0040	*Klebsiella pneumoniae*	SAMN45881404	This study
2401G0051	*Klebsiella pneumoniae*	SAMN45881453	This study
2401G0058	*Klebsiella pneumoniae*	SAMN45881428	This study
2401G0061	*Klebsiella pneumoniae*	SAMN45881409	This study
2401G0063	*Klebsiella pneumoniae*	SAMN45881445	This study
2401G0064	*Klebsiella pneumoniae*	SAMN45881410	This study
2401G0065	*Klebsiella pneumoniae*	SAMN45881400	This study
2401G0066	*Klebsiella pneumoniae*	SAMN45881403	This study
2401G0068	*Klebsiella pneumoniae*	SAMN45881435	This study
2401G0069	*Klebsiella pneumoniae*	SAMN45881438	This study
2401G0071	*Klebsiella pneumoniae*	SAMN45881455	This study
2401G0073	*Klebsiella pneumoniae*	SAMN45881436	This study
2401G0074	*Klebsiella pneumoniae*	SAMN45881405	This study
2401G0075	*Klebsiella pneumoniae*	SAMN45881440	This study
2401G0076	*Klebsiella pneumoniae*	SAMN45881447	This study
2401G0080	*Klebsiella pneumoniae*	SAMN45881422	This study
2401G0081	*Klebsiella pneumoniae*	SAMN45881429	This study
2401G0093	*Klebsiella pneumoniae*	SAMN45881430	This study
2401G0094	*Klebsiella pneumoniae*	SAMN45881473	This study
2401G0095	*Klebsiella pneumoniae*	SAMN45881423	This study
2401G0097	*Klebsiella pneumoniae*	SAMN45881418	This study
2401G0100	*Klebsiella pneumoniae*	SAMN45881443	This study

2401G0101	*Klebsiella pneumoniae*	SAMN45881408	This study
2401G0104	*Klebsiella pneumoniae*	SAMN45881411	This study
2401G0111	*Klebsiella pneumoniae*	SAMN45881471	This study
2401G0113	*Klebsiella pneumoniae*	SAMN45881474	This study
2401G0121	*Klebsiella pneumoniae*	SAMN45881493	This study
2401G0143	*Klebsiella pneumoniae*	SAMN45881482	This study
2401G0144	*Klebsiella pneumoniae*	SAMN45881465	This study
2401G0147	*Klebsiella pneumoniae*	SAMN45881464	This study
2401G0150	*Klebsiella pneumoniae*	SAMN45881461	This study
2401G0151	*Klebsiella pneumoniae*	SAMN45881407	This study
2401G0153	*Klebsiella pneumoniae*	SAMN45881475	This study
2401G0157	*Klebsiella pneumoniae*	SAMN45881452	This study
2401G0160	*Klebsiella pneumoniae*	SAMN45881419	This study
2401G0166	*Klebsiella pneumoniae*	SAMN45881479	This study
2401G0170	*Klebsiella pneumoniae*	SAMN45881442	This study
2401G0172	*Klebsiella pneumoniae*	SAMN45881481	This study
2401G0033	*Escherichia coli*	SAMN45881439	This study
2401G0035	*Escherichia coli*	SAMN45881446	This study
2401G0041	*Escherichia coli*	SAMN45881470	This study
2401G0048	*Escherichia coli*	SAMN45881444	This study
2401G0053	*Escherichia coli*	SAMN45881426	This study
2401G0055	*Escherichia coli*	SAMN45881454	This study
2401G0056	*Escherichia coli*	SAMN45881427	This study
2401G0062	*Escherichia coli*	SAMN45881480	This study
2401G0070	*Escherichia coli*	SAMN45881450	This study
2401G0072	*Escherichia coli*	SAMN45881437	This study
2401G0077	*Escherichia coli*	SAMN45881434	This study
2401G0083	*Escherichia coli*	SAMN45881468	This study
2401G0084	*Escherichia coli*	SAMN45881456	This study
2401G0088	*Escherichia coli*	SAMN45881467	This study
2401G0091	*Escherichia coli*	SAMN45881431	This study
2401G0099	*Escherichia coli*	SAMN45881459	This study
2401G0106	*Escherichia coli*	SAMN45881472	This study
2401G0109	*Escherichia coli*	SAMN45881413	This study
2401G0125	*Escherichia coli*	SAMN45881466	This study
2401G0142	*Escherichia coli*	SAMN45881477	This study
2401G0146	*Escherichia coli*	SAMN45881492	This study
2401G0148	*Escherichia coli*	SAMN45881463	This study
2401G0152	*Escherichia coli*	SAMN45881476	This study

2401G0161	*Escherichia coli*	SAMN45881416	This study
2401G0162	*Escherichia coli*	SAMN45881451	This study
2401G0164	*Escherichia coli*	SAMN45881494	This study
2401G0165	*Escherichia coli*	SAMN45881478	This study
2401G0177	*Escherichia coli*	SAMN45881469	This study

All listed sequence accession numbers are available in the NCBI database (U.S. National Library of Medicine).

TABLE S2 Comparison of detection results across assays for six clinical GN-PBC samples with multiple β-lactam resistance genes identified.

Sample	Organism	Detection of *bla* genes encoding the indicated β-lactamases by assay		
		**GNR microchip**	**WGS reference**	**BCID2 panel**
#1	*K. pneumoniae*	CMY-2-like, CTX-M-1/9 group, KPC, VIM	CMY-16, CTX-M-15, KPC-3, VIM-1	CTX-M, KPC, VIM
#2	*E. coli*	CMY-2-like, CTX-M-1/9 group	CMY-2, CTX-M-15	CTX-M
#3	*E. coli*	CMY-2-like, CTX-M-1/9 group	CMY-147, CTX-M-15	CTX-M
#4	*A. baumannii*	OXA-23-like, NDM	OXA-23, NDM-1	NDM
#5	*E. cloacae*	CTX-M-1/9 group, SHV-ESBL, VIM	CTX-M-3, SHV-12, VIM-1	CTX-M, VIM
#6	*C. freundii*	CMY-2-like, VIM	CMY-150, VIM-1	VIM

GN-PBC, Gram-negative-positive blood culture; GNR, Gram-negative resistance; WGS, whole-genome sequencing; BCID2, Blood Culture Identification 2.
